# SLEEP TIGHT, STAY HEALTHY? MULTIDIMENSIONAL SLEEP HEALTH AND CHRONIC CONDITION DEVELOPMENT OVER ONE DECADE

**DOI:** 10.1093/geroni/igad104.1828

**Published:** 2023-12-21

**Authors:** Claire Smith, Soomi Lee

**Affiliations:** University of South Florida, Tampa, Florida, United States; The Pennsylvania State University, University Park, Pennsylvania, United States

## Abstract

Chronic conditions affect half of adults and are the leading cause of death in the United States. Healthy sleep is a modifiable risk factor, but greater information is needed about specific sleep experiences linked to chronic condition development. The present study aims to (1) identify within-person patterns of various sleep experiences (i.e., sleep health phenotypes describing sleep duration, regularity, sleep onset latency, insomnia symptoms, daytime tiredness, and nap frequency) and (2) understand the connection between transitions in sleep health phenotypes and chronic condition development. We use self-report data from a national sample of adults (N=5,026) taken from the Midlife in the United States (MIDUS) study across two waves (II and III) separated by about ten years. Latent transition analysis revealed four sleep phenotypes: good sleepers (optimal sleep across dimensions), insomnia sleepers (short duration, long sleep onset latency, high daytime tiredness, frequent insomnia symptoms), weekend catch-up sleepers (long sleep duration on weekends compared to weekdays), and nappers (frequent naps). For cardiovascular conditions, consistent insomnia sleepers (i.e., insomnia sleeper -> insomnia sleeper) were at 72% higher risk (CI:[1.04, 2.82]) than consistently good sleepers. For diabetes, consistent insomnia sleepers again were at higher risk (188%; CI:[1.72,4.79]) as were consistent nappers (128%; CI:[1.26,4.09]) and weekend catch-up sleepers -> nappers (137%; CI:[1.21,4.60]). For cancers, only nappers -> insomnia sleepers exhibited higher risk (45%; CI:[1.16,1.83]). We newly identify two sleep health phenotypes (napper and, especially, insomnia sleeper) as risks for developing life-threatening chronic conditions.

